# Retrospective study of the morphology of third maxillary molars among the population of Lower Silesia based on analysis of cone beam computed tomography

**DOI:** 10.1371/journal.pone.0299123

**Published:** 2024-02-23

**Authors:** Anna Olczyk, Barbara Malicka, Katarzyna Skośkiewicz-Malinowska

**Affiliations:** Department of Conservative Dentistry with Endodontics, Wroclaw Medical University, Wrocław, Poland; University of Puthisastra, CAMBODIA

## Abstract

**Introduction:**

Understanding the anatomy of root canal systems and being aware of their variations is crucial for successful endodontic treatment. Specifically, the intricate and diverse nature of the root anatomy in maxillary third molars poses a significant challenge for dental clinicians. The study analyzed the morphology of the root canal system in maxillary third molars among residents of the Lower Silesia region in Poland using cone beam computed tomography (CBCT).

**Material and methods:**

This retrospective cross-sectional imaging study was conducted at the X-Ray Diagnostics Laboratory of the Academic Dental Polyclinic of Wroclaw Medical University, Poland. The study evaluated 316 CBCT scans and included 196 maxillary third molars. They were obtained from 118 females and 78 males over the age of 18, in the period from January to April 2022 (three consecutive months). The number of roots, root canals, and root canal configurations according to Vertucci’s classification were analyzed.

**Results:**

Three-rooted maxillary molars were found most often (64.29%), followed by single-rooted (24.49%), two-rooted (7.65%), and four-rooted (3.57%) teeth. Among all the roots examined, Vertucci Type I root canals were the most prevalent. Our research found that single-rooted forms (40 teeth, 33.89% vs. 8 teeth, 10.26%, p = 0.0013) of maxillary third molars were significantly more common in females than in males. However, three-rooted forms (62 teeth, 79.49% vs. 64 teeth, 54.24%, p = 0.0013) of maxillary third molars were significantly more common in males than in females.

**Discussion:**

In the Lower Silesia region, the typical maxillary third molar in the Polish population has a three-rooted structure with Vertucci Type I root canal configuration. We noted a sex-dependent correlation in maxillary third molar morphology, with single-rooted forms more prevalent in women and three-rooted forms in men.

## Introduction

Familiarity with the anatomy of root canals and awareness of their variability determines the outcome of endodontic treatment. The anatomy of maxillary third molars is particularly complex and distinct, and therefore poses a challenge to clinicians [1, 2]. Knowledge of root canal morphology is indispensable for successful endodontic treatment, and the anatomical variability of root canal systems is a substantial therapeutic challenge. Proper diagnosis of the root canal system is necessary to successfully conduct endodontic treatment, and failure of root canal treatment can result from many factors, such as not detecting or omitting canals due to their complex morphology. Untreated canals are reservoirs of bacteria that ultimately lead to complications [3–8]. Various techniques have been used to assess root canal morphology in vivo and in vitro studies: staining techniques, ground teeth sections, stereomicroscopy, scanning electron microscopy, optical coherence tomography, endoscopy, conventional two-dimensional (2D) periapical radiographs, cone beam computed tomography (CBCT), and micro-computed tomography (micro-CT). CBCT is currently the preferred method of examination, providing three-dimensional images of the teeth and surrounding structures in three orthogonal planes: axial, coronal, and sagittal, enabling in vivo examination [3–5, 7, 9–16]. CBCT examination eliminates the overlapping of adjacent structures, and its three-dimensional nature makes it superior to conventional two-dimensional radiography. CBCT is considered to be an effective diagnostic tool that can produce results similar to using a modified tooth staining technique to identify differences in root canal morphology [3–5, 7]. An alternative methodology that facilitates three-dimensional (3D) imaging is X-ray micro-computed tomography (micro-CT), which excels in capturing images at a considerably reduced scale while preserving a markedly enhanced resolution. In contrast, cone beam computed tomography (CBCT) remains the primary method of rapid image acquisition, characterized by reduced radiation exposure and cost-effectiveness [17].

**Fig 1:**
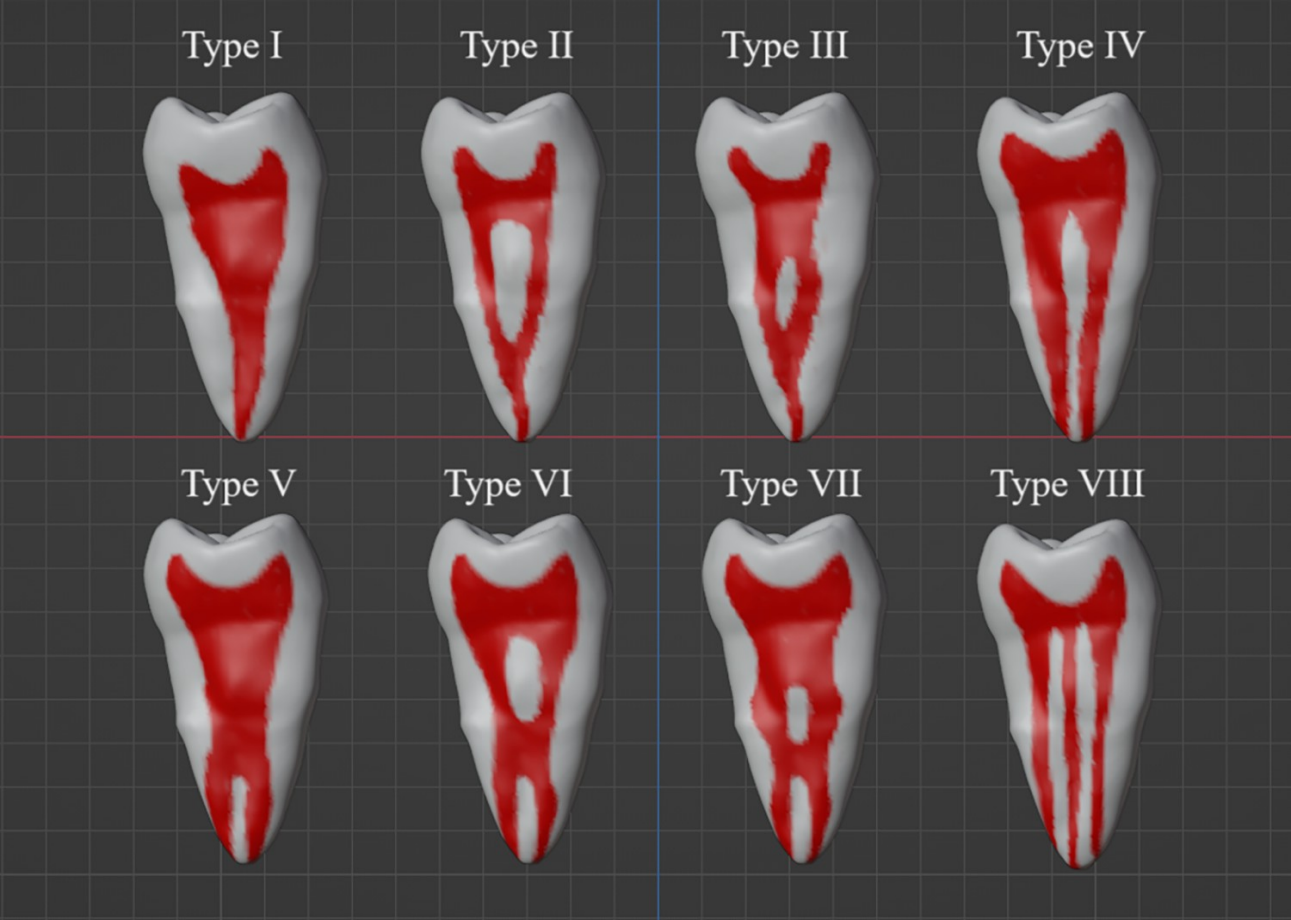
Vertucci’s classification.

Many classifications have been introduced based on canal morphology. Weine [18] classified the network of root canals, dividing it into four specific forms in any root: Type I (1–1) with a single canal from the pulp chamber to the apex, Type II (2–1) with two individual canals from the pulp chamber ending as one, short of the apex, Type III (2–2) with two separate canals from the pulp chamber to the root apex, and Type IV (1–2) with one canal starting in the pulp chamber and dividing into two canals near the root apex. In 1974, Vertucci [19] (Fig 1) divided root canal structures into eight forms based on the anatomy of the human maxillary second premolar: Type I (1–1) with a single canal from the pulp chamber to the apex; Type II (2–1) with two separate canals leaving the pulp chamber but joining short of the apex to form one canal; Type III (1-2-1) with one canal leaving the pulp chamber, dividing into two, and merging again to end as one canal; Type IV (2–2) with two canals from the pulp chamber to the apex; Type V (1–2) with one canal leaving the pulp chamber and dividing into two separate canals with separate apical foramina; Type VI (2-1-2) with two separate canals leaving the pulp chamber, merging into one in the body of the root and redividing into two separate canals short of the apex; Type VII (1-2-1-2) with one canal leaving the pulp chamber, dividing into two, then rejoining and dividing again into two canals with two separate foramina; and Type VIII (3–3) with three canals from the pulp chamber to the apex. A study of a Burmese population [20] added new types of canals to the list: Type (1-3-1), Type (2-1-2-1), Type (2–3), Type (4–1), Type (3–2), Type (3–4), and Type (4–4). Another study on a Turkish population introduced 14 different types of root canals, which led to the creation of an expanded classification of root canal types proposed by Vertucci, resulting in a total number of 23 root canal types: 1-VIII–from Vertucci and IX to XXIII from Sert and Bayirli [21]: IX (1–3), X (1-2-3-2), XI (1-2-3-4), XII (2-3-1), XIII (1-2-1-3), XIV (4–2), XV (3–2), XVI (1-3-1), XVII (3–1), XVIII (2-1-2-1), XIX (4), XX (4–1), XXI (5–4), XXII (3–4), and XXIII (2–3).

In 2014, Leoni et al. [22] were studying the anatomy of mandibular incisors and found eight new types of canal configurations:1-2-3-1, 1-2-3-2-3, 1-2-3-2-1, 1-3-2-1-2-1-2-1, 1-2-1-2-3-2-1-2-2-1, 1-2-1-2-3-2-3-2-1, 1-2-1-2-3-2-1-2-1-2-1, and 1-2-3-2-3-2-3-2-1-2-1, while Ordinola, Zapata et al. [23] one year later, were studying the anatomy of the mandibular first premolar and discovered new canal types: 1-2-3, 1-2-3-2, 1-2-3-2-3 and 1-2-1-2-4. In 2017, Ahmed et al. [24] created a new classification that provides an unlimited number of root canal types described through coding rather than creating new types. However, considering that the most widespread classification is the one proposed by Vertucci, which covers all types of canals encountered in the presented study, the authors chose to use Vertucci’s classification.

The study aimed to analyze the morphology of the root canal systems of maxillary third molars (including root number, canal number, and the configurations of those canals) in the population of the region of Lower Silesia (Poland) with the use of cone beam computed tomography (CBCT). The following research questions are planned to be answered:

1. How many roots and root canals were observed in the maxillary third molars within the study population?
2. What was the most prevalent configuration of root canals in maxillary third molars within the studied population?

Additionally, the hypothesis will be tested that there is no difference between sex regarding the number of root in maxillary third molars within the studied population.

## Material and methods

**Fig 2:**
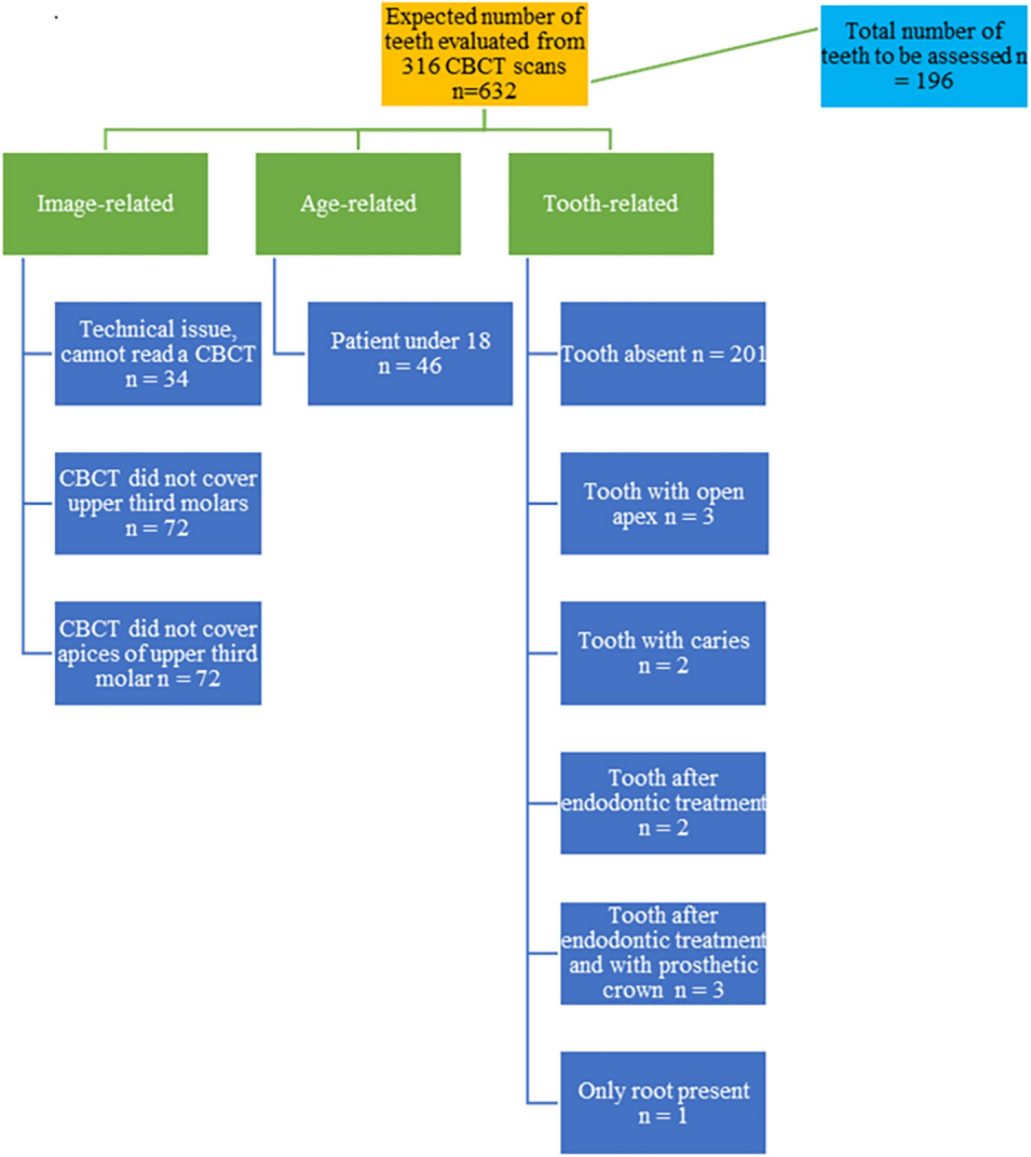
Exclusion criteria.

This retrospective cross-sectional imaging study was conducted at the X-Ray Diagnostics Laboratory of the Academic Dental Polyclinic of Wroclaw Medical University, Poland, with the use of a CBCT scanner–Rayscan α-Plus 130 (Rayscan 700) (Ray Europe GmbH, Seoul, South Korea), with the field of view varying from 3x4 cm to 13x10 cm, 60–90 kV, 4–17 mA, scan time of 14 seconds, voxel size of 70 μm, 160 μm, 200 μm or 280 μm, and spaces between slices of 0.07–3mm was used. For the purpose of the study, only full-mouth CBCTs were considered, with a voxel size of 160 μm, spaces between slices 0,16 mm, field of view 13x10 cm, 90 kV, 4mA and images taken for various dental diagnostics, and accessed via Rayscan SMART Dent (Ray Co., Ltd, Seoul, South Korea), and OnDemand3DApp software (Cyber Med, Seoul, South Korea). The study covered the period from January to April 2022. The study protocol was approved by the Bioethics Committee of Wroclaw Medical University (approval no. KB 206/2022) under the Declaration of Helsinki. Data for research purposes were accessed on May 2, 2022. The process of obtaining consent was conducted in a documented and informed manner. The entirety of the data underwent a comprehensive anonymization process, ensuring the complete removal of personally identifiable information. The authors did not have access to information that could identify individual participants during or after data collection. A total of 316 cone beam computed tomography scans were analyzed. All scans were evaluated separately by two endodontists (with 5 and 20 years of professional experience), and any disagreement was discussed until a consensus was reached. All the images were analyzed using a Beacon Display C25W screen (Shenzhen Beacon Display Technology Co., Ltd.) with a resolution of 1920 x 1080 pixels. Scans were evaluated by zooming, magnification, contrast, and brightness adjustment in a low-lighted room. No more than 10 scans per session were assessed to avoid visual fatigue. Of a potential number of 632 teeth, 196 maxillary third molars meeting the inclusion criteria were included in the study. Teeth eligible for the study belonged to patients over 18 years of age, of both sexes. Teeth included in the study had to have a closed apex, no caries or fillings, no prosthetic crowns or post-core inlays, no periapical changes, calcifications, or resorptions; the teeth could not have been endodontically treated before; CBCT images could not show artifacts (Fig 2). Teeth in mesiolingual and distolingual rotation were included in the study as well as impacted teeth were included in the study. Tooth anatomy was assessed according to the following parameters: the number of roots, number of canals, and root canal configurations. Root canal morphology was classified according to Vertucci [19].

The evaluation process proceeded with the training and calibration of both observers. Sample CBCT scans representing all eight types of Vertucci’s classification were assessed. The kappa statistic (Cohen’s kappa) was used to determine the interobserver agreement value. The kappa value was 92.3%, which is considered excellent [25].

### Statistical analysis

The results were submitted to statistical analysis with Statistica 13 (2017) software (TIBCO Software Inc., USA) by the Statistical Analysis Center of Wroclaw Medical University. The analysis included the number of roots (Table 1), the number of roots depending on sex (Tables 2 and 3), and Vertucci’s classification (Table 4 and 5). Table 2 shows the chi-squared and p-values comparing the frequency of each tooth type depending on the patient’s sex. The chi-squared test provided a significance level below 0.0125 (Table 2). The Bonferroni correction was used to control Type I error, i.e., incidental finding of significant differences. The threshold for statistical significance is 0.0125. In Tables 1 and 5, the confidence intervals of the observed rates were calculated.

**Table 1:** Frequency (%) of roots in maxillary third molars with 95% confidence intervals (CI).

Number of roots	Total			
	n	%	Proportion	95% CI
1	48	24.49%	0.24	(0.18; 0.31)
2	15	7.65%	0.08	(0.04; 0.11)
3	126	64.29%	0.64	0.58; 0.71)
4	7	3.57%	0.04	(0.01; 0.06)
Total number	196	100.00%	-	-

**Table 2:** Frequency (%) of roots in maxillary third molars depending on sex.

Number of roots	Female		Male		Chi-square statistic	P-value
	n	%	n	%		
1	40	33.89	8	10.26	15.66	**0.0013**
2	10	8.48	5	6.41		
3	64	54.24	62	79.49		
4	4	3.39	3	3.84		
Total number	118	100	78	100		

Threshold of statistical significance p < 0.0125

**Table 3:** A post hoc test of the differences in the frequency of roots between the sexes (females vs. males) in maxillary third molars.

Comparison	Chi-square statistic	P-value
**1-root vs. others**	**14.19**	**<0.0002**
2-root vs. others	0.28	0.5946
**3-root vs. others**	**13.04**	**0.0003**
4-root vs. others	-*	1.0000

*—no value because Fisher’s exact test was used

Threshold of statistical significance p < 0.0125 (with the Bonferroni correction)

**Table 4:** Vertucci’s canal type in the study population.

		Vertucci’s canal types							
Number of roots	Root	I	II	III	IV	V	VI	VII	VIII
		n (%)	n (%)	n (%)	n (%)	n (%)	n (%)	n (%)	n (%)
**1**	-	**38 (79.17)**	0	1 (2.08)	4 (8.33)	1 (2.08)	0	0	4 (8.33)
	female/male	**34**/4	0/0	1/0	3/1	0/1	0	0	2/2
**2**	B	**13 (86.67)**	0	0	2 (13.33)	0	0	0	0
	female/male	**8**/5	0/0	0/0	2/0	0/0	0/0	0/0	0/0
	P	**15 (100)**	0	0	0	0	0	0	0
	female/male	**10**/5	0/0	0/0	0/0	0/0	0/0	0/0	0/0
**3**	MB	**99 (78.6)**	0	1 (0.08)	24 (19.05)	2 (1.6)	0	0	0
	female/male	**53**/46	0/0	0/1	10/14	1/1	0/0	0/0	0/0
	DB	**125 (99.2)**	1 (0.08)	0	0	0	0	0	0
	female/male	**63**/62	1/0	0/0	0/0	0/0	0/0	0/0	0/0
	P	**125 (99.2)**	1 (0.08)	0	0	0	0	0	0
	female/male	**63**/62	1/0	0/0	0/0	0/0	0/0	0/0	0/0
**4**	MB	**7 (100)**	0	0	0	0	0	0	0
	female/male	**4**/3	0/0	0/0	0/0	0/0	0/0	0/0	0/0
	MB2	**7 (100)**	0	0	0	0	0	0	0
	female/male	**4**/3	0/0	0/0	0/0	0/0	0/0	0/0	0/0
	DB	**7 (100)**	0	0	0	0	0	0	0
	female/male	**4**/3	0/0	0/0	0/0	0/0	0/0	0/0	0/0
	P	**7 (100)**	0	0	0	0	0	0	0
	female/male	**4**/3	0/0	0/0	0/0	0/0	0/0	0/0	0/0

**Table 5:** Proportions (relative to the sum of canal types) and their 95% confidence intervals (CI).

Number of roots	Root	Total number	Vertucci’s canal types					
			Type I		Type IV		Type VIII	
			Proportion	95% CI	Proportion	95% CI	Proportion	95% CI
**1**	-	48	0.79	(0.674; 0.905)	0.08	(0.003; 0.157)	0.08	(0.003; 0.157)
**2**	B	15	0.87	(0.700; 1.000)	0.13	(0.000; 0.300)	-	-
	P	15	1.00				-	-
**3**	MB	126	0.79	(0.719; 0.861)	0.19	(0.122; 0.258)	-	-
	DB	126	0.99	(0.973; 1.000)	-	-	-	-
	P	126	0.99	(0.973; 1.000)	-	-	-	-
**4**	MB	7	1.00	-	-	-	-	-
	MB2	7	1.00	-	-	-	-	-
	DB	7	1.00	-	-	-	-	-
	P	7	1.00	-	-	-	-	-

All available (n = 316) scans were analyzed, and the power of the analysis of maxillary third molars that met the inclusion criteria (n = 196) was calculated a posteriori using the “power.chisq.test” function from the “DescTools” R-package. When performing the chi-squared test from 2x4 tables, the sample size of n = 196 makes it possible to detect Cohen’s effect size “w” equal to 0.24 at the power of 0.80, an alpha level of 0.05 (in a two-tailed test). According to Cohen’s classification, w = 0.24 indicates an effect size between “small” (0.1) and “medium” (0.3).

The study was performed according to the Anatomical Quality Assurance (AQUA) checklist [26].

## Results

### 1. Study group characteristics

After analyzing 316 cone beam computed tomography scans from the potential number of 632 teeth, 196 maxillary third molars were qualified for examination, belonging to 118 (60.2%) females and 78 (39.8%) males. The main factors preventing the inclusion of teeth in the study group were the absence of maxillary third molars in a patient or a CBCT examination without the third molar or its apex. The scheme of the study’s inclusion and exclusion criteria is presented in Fig 2.

### 2. Number of roots

Among the 196 analyzed teeth scans included in the study, three-rooted teeth were the most prevalent (64.29%, n = 126), while single-rooted teeth (24.49%, n = 48), double-rooted teeth (7.65%, n = 15), and four-rooted teeth (3.57%, n = 7) occurred less frequently (Table 1).

In addition, the frequency of occurrence of individual maxillary third molar types (with different numbers of roots) depending on the patient’s sex was analyzed (Tables 2 and 3). Our research found that single-rooted forms (40 teeth, 33.89% vs. 8 teeth, 10.26%, p = 0.0013) of maxillary third molars were significantly more common in females than in males. However, three-rooted forms (62 teeth, 79.49% vs. 64 teeth, 54.24%, p = 0.0013) of maxillary third molars were significantly more common in males than in females (Table 2). The results have been confirmed by a post hoc test (Table 3). A similar relationship was observed for fused teeth (18 teeth, 75%, vs. 6 teeth, 25%). Among two-rooted teeth, fused teeth accounted for 20%, of which 66.67% belonged to females and 33.33% to males. Among three-rooted teeth, fused teeth accounted for 15.08%, of which 73.68% belonged to females and 26.32% to males. Among four-rooted teeth, two fused teeth were found, both in women.

### 3. Number of canals and root canal configurations (Table 4)

#### 3.1 Single-rooted teeth

Single-rooted teeth most often (38 teeth, 79.17%) had Type I root canals according to Vertucci’s classification (Table 5). Vertucci Types II, VI, and VII were not found in the study group, while Types IV (8.33%) and VIII (8.33%) occurred sporadically, as did Type III (2.08%) and Type V (2.08%).

#### 3.2 Two-rooted teeth

Among two-rooted forms, Type I root canals, according to Vertucci’s classification, were most often observed in buccal (B) (86.67%) and palatal (P) (100%) roots (Table 5). In buccal roots, Vertucci Type IV was additionally found in 13.33% of cases; such a configuration was observed in two-rooted teeth with fused roots in women.

#### 3.3 Three-rooted teeth

Three-rooted teeth were most often characterized by Type I root canals, according to Vertucci’s classification, in the palatal (P) (99.2%), mesiobuccal (MB) (78.6%), and distobuccal (DB) (99.2%) roots (Table 5). In the mesiobuccal root, Type IV configuration was additionally observed in 19.05% of cases. In the studied group of three-rooted teeth, there were also teeth with fused roots, and this fusion most often (11 teeth) involved a combination of all roots (MB, DB, and P) or MB and DB roots (4 teeth) and DB and P roots (1 tooth).

#### 3.4 Four-rooted teeth

In the studied group, Type I root canals, according to Vertucci’s classification, were found in each of the four canals (Table 5). In this group, root fusion was also observed–the fusion of mesiobuccal first (MB1) and mesiobuccal second (MB2) roots or fusion of MB1, MB2, and DB roots.

## Discussion

Numerous papers have been published on the anatomy of molars [27–30] but the number of works discussing the anatomy of the third molars of the maxilla is small. Therefore, attempts were made to systematize the knowledge about the structure of these teeth in the Polish population. A retrospective study of the morphology of the maxillary third molars in the population of Lower Silesia was conducted based on the analysis of cone beam computed tomography scans, which found that maxillary third molars have one to four roots, with the highest occurrence of three-rooted forms (64.29%) with three canals of Type I configuration according to Vertucci’s classification (85.71%). The results were consistent with the results of earlier studies on the anatomy of maxillary third molars [20, 31–37], where it was found, as in the authors’ research, that these teeth most often had three roots and three canals. According to Vertucci’s classification, MB, DB, and P roots were most often Type I in the three-rooted maxillary third molars. The authors point out that differences in dental anatomy variation could also be associated with age, sex, and ethnic differences between the study populations [9, 27, 32, 38–43]. Our research found that single-rooted forms (40 teeth, 33.89%, vs. 8 teeth, 10.26%, p = 0.0013) of maxillary third molars were significantly more common in females than in males. However, three-rooted forms (62 teeth, 79.49%, vs. 64 teeth, 54.24%, p = 0.0013) of maxillary third molars were significantly more common in males than in females. The results cannot be compared with the results of other studies due to the lack of data on the anatomical structure of maxillary third molars dependent on sex. The research conducted by the authors leads to the hypothesis that both fused roots and single-rooted teeth are more common in women, and perhaps the reason for this may be dental arches, which are smaller in women than in men. A study of mandibular first premolars showed a significant tendency toward single-rooted versus double-rooted teeth in females [44]. Females generally have smaller dental arches [45]. Another study that analyzed root fusion in molars showed that root fusion was approximately 5% more common in females than in males, and molar root fusion was observed in females about 13% more often than in males [46]. Ahmad et al.’s research on third molars indicated that the incidence of root fusion was more pronounced in females compared to males, with a statistically significant difference specifically in mandibular molars [47]. More research should be conducted on the cause of the greater prevalence of root fusion in females. Considering the differences in sample origin, it should be noted that the available studies were conducted mainly in India [31, 32], Myanmar [20], Thailand [33], China [34] and Turkey [35] which is why in the future, studies of the morphology of maxillary third molars covering other continents and populations should be conducted in order to verify the hypothesis of anatomical differences between populations.

At the same time, one cannot ignore the fact that our research and the cited studies [20], [31–34, 36] differed in methodology (techniques used to identify canal configurations). The authors’ research was retrospective and evaluated CBCT scans, while the other studies were performed on extracted teeth, and the assessment of tooth anatomy was carried out based on the CBCT image [31] or the method of canal staining [20, 32–36]. Literature evidence suggests that while the tooth-clearing technique and canal staining used to be the gold standard procedure, CBCT is considered the new standard [48]. CBCT has been found to be equally accurate and effective both in vitro and in vivo, offering the benefit of being noninvasive [3–5, 7].

CBCT exhibits several advantages when juxtaposed with alternative radiographic methodologies. In contrast to conventional radiography, CBCT enables the visualization of dental structures in three-dimensional spatial dimensions as opposed to the conventional two-dimensional representation. An additional consideration pertains to the necessity for dental practitioners to acquire proficiency in interpreting CBCT images emanating from diverse software providers, thereby necessitating a temporal investment. The efficacy of CBCT is contingent upon the practitioner’s adeptness in navigating the nuances of the technique and discerning the diagnostic value inherent in the acquired images [49]. The radiation dosage associated with an individual conventional X-ray commences at 0.5 μSv, whereas for CBCT, it initiates at 20 μSv. Anticipated advancements in technology are poised to result in a reduction of radiation doses, and the burgeoning prevalence of CBCT scanners is expected to concomitantly precipitate a decline in their market prices. Endodontic diagnosis and treatment planning have taken a giant leap forward due to the introduction of CBCT in dentistry [50]. While conventional 2D radiographs remain the most cost-effective and routine method to evaluate a patient’s dentition, their diagnostic potential is limited. The manipulation of 3D images offered by CBCT provides better insight into diagnostic dilemmas and complex treatment decisions. Despite the advantages of CBCT imaging, it should be used as a complement to 2D radiography, not as a replacement. The principle of ALARA (in which patients should be exposed to radiation “as low as reasonably achievable”) still applies to this technology. CBCT should not be used routinely in the absence of clinical signs or symptoms that necessitate a more in-depth view of the tooth and surrounding structures. In other words, if conventional 2D radiography is sufficient, then a pretreatment CBCT scan is unnecessary. However, if more information is needed to make an accurate diagnosis, a 3D CBCT image is justified and highly beneficial, as shown through several case examples presented in this article [51].

The study presented here is a supplement to the information about the morphology of teeth contained in the works of Olczak et al. [4, 5]. They analyzed the number of roots and root canals in maxillary first and second molars (2017) and maxillary first premolars (2022) in the Polish population [4, 5]. Olczak and Pawlicka reported significant differences in the distribution of the number of roots and root canals in maxillary first and second molars. They found that all maxillary first molars had three roots (100%), but maxillary second molars had three roots (91.8%), two roots (5.8%), or one root (2.4%). According to our findings, three-rooted teeth were the most prevalent (64.29%), while single-rooted teeth (24.49%), double-rooted teeth (7.65%), and four-rooted teeth (3.57%) occurred less frequently.

Olczak and Pawlicka [4] also observed differences in the number of root canals. Maxillary first molars had four root canals (59.5%) or three root canals (40.5%), while maxillary second molars most often had three root canals (70%), rarely four canals (23.2%), two canals (3.9%), one canal (1%), or C-shaped canals (1.9%). More than three root canals in maxillary first molars occurred at various frequencies depending on the study population: lower in the Polish (59.5%) [4] and Greek populations (56.1%) [52], but higher in the Yemeni (82.6%) [53], Iranian (89.9%) [54], Emirati (80.1%) [55], Taiwanese (79.3%) [56], Saudi (70.6%) [57], and Southern Chinese (68.1%) populations [58]. Additional canals in the mesiobuccal roots were observed more frequently in maxillary first molars (59.5%) than in second molars (23.2%) [4]. In our study, Type IV configuration in the mesiobuccal root (MB1, MB2) was observed in 19.05% of cases. Stropko [59] observed the MB2 canal in 73.2% of first molars, 50.7% of second molars, and 20.0% of third molars. In the Burmese population, the prevalence of mesiobuccal roots of maxillary molars with two canals was as follows: first molars– 68%, second molars– 49%, and third molars– 39% [20]. The prevalence of the MB2 canal in the Saudi population was lower–with 46.7% in first molars and 17.7% in second molars. Additionally, the authors observed that the MB2 canal was more common in females [60]. In the Iraqi population, the MB2 canal was found in 53.78% of first molars, and the MB2 canal was detected significantly more often in males than in females, but the study found no correlation with age [61].

A review of the root anatomy of maxillary second molars [62] indicated that the three-rooted form was the most common, while the four-rooted anatomy was the least prevalent. In the Polish population, maxillary second molars had three roots (91.8%), two roots (5.8%), or one root (2.4%) [4]. In the Saudi population, the second molar most often had three roots (93.0%), and only 4.0% had two roots. Additionally, the prevalence of MB2 was found to be 49.7% [63]. Similar findings in the Saudi population were reported by Alamri et al. [64]–the majority of maxillary second molars (92%) had three roots, while two roots (6.6%), four roots (1.1%), and one root (0.3%) were found less frequently. The authors additionally reported a significant correlation between sex and the number of root canals–females were more likely to have teeth with two canals, while males had four canals [64].

The occurrence of differences in anatomical structure should always be taken into account during endodontic treatment, however, research results indicate that the third maxillary molars are most often three-rooted teeth with three canals of Vertucci Type I canal configuration, which should be taken into account by dentists during endodontic treatment of patients.

A few limitations of this study must be considered. The relatively small study group was one of them. The study included a total of 316 cone beam tomography scans performed from January to April 2022 (three consecutive months). After analyzing 316 cone beam computed tomography scans from the potential number of 632 teeth, 196 maxillary third molars were qualified for examination. The main factor preventing the inclusion of teeth in the study group was the absence of maxillary third molars in a patient (n = 201). It should be emphasized that tooth agenesis (the congenital absence of one or more teeth) is an important factor that must be taken into account when analyzing third molars [65]. Based on data from the systematic review and meta-analysis of Carter et al. [66], the worldwide rate of agenesis was found to be 22.63% (95% confidence interval = 20.64% to 24.76%), although the estimates ranged from 5.32% to 56.0%. Therefore, this characteristic will have a significant impact on the sample size. A certain methodological constraint may be that the research was conducted in one center–the X-Ray Diagnostics Laboratory at the Academic Dental Polyclinic, Wroclaw Medical University. According to the Nomenclature of Territorial Units for Statistics (NUTS), as of January 2018, there are seven units in Poland–NUTS 1 macroregions, including the southwestern macroregion (PL5), which includes the Lower Silesia Province (PL51) with its capital in Wroclaw (PL514). The X-Ray Diagnostics Laboratory at the Academic Dental Polyclinic is the central unit providing services to all departments of the Academic Dental Polyclinic of Wroclaw Medical University, which enabled access to a large and, at the same time, diverse group of patients, giving a representative picture of the Polish population. It is likely that multicenter research, including other centers in Poland and Europe, would better reflect the general population, allowing for more conclusive results encompassing a broad geographic scope and ethnic characteristics. Another limitation of the present study is the choice of the technique used to assess root canal morphology–CBCT, which could lead to potential bias. Micro-CT produces images on a much smaller scale and with greater resolving power. However, CBCT was selected due to the radiological protection of patients and the lower examination cost.

## Conclusions

In the Polish population of the Lower Silesia region, the prevailing maxillary third molar anatomy is a three-rooted tooth with three canals of Vertucci Type I configuration, and a sex-related association is observed, with single-rooted forms more prevalent in women and three-rooted forms in men, providing valuable insights for dental clinicians, while the use of CBCT as a noninvasive diagnostic tool can enhance successful endodontic treatment.

## Supporting information

S1 Checklist RECORD statement—checklist of items that should be included in reports of retrospective studies. (PDF)

S1 File Upper third molars data table Excel file. (XLSX)
