# Supplementary material for: Retrospective study of the morphology of third maxillary molars among the population of Lower Silesia based on analysis of cone beam computed tomography
Source: PLoS One. 2024 Feb 23;19(2):e0299123. doi: 10.1371/journal.pone.0299123 (PMC10890766; doi:10.1371/journal.pone.0299123)
Supplement: S1 Checklist — (PDF) [file pone.0299123.s001.pdf]

**RECORD Statement—checklist of items that should be included in reports of retrospective studies**

| | Item No | Recommendation | Page & Line # |
|---|---|---|---|
| **Title and abstract** | 1 | (*a*) Indicate the study's design with a commonly used term in the title or the abstract | 1 #1 |
| | | (*b*) Provide in the abstract an informative and balanced summary of what was done and what was found | 2, #25-51 |
| | | *(c)* The type of data used should be specified in the title or abstract. When possible, the name of the databases used should be included. If applicable, the geographic region and time frame within which the study took place should be reported in the title or abstract. If linkage between databases was conducted for the study, this should be clearly stated in the title or abstract. | 1, #1-5, 2, #32-38, |
| **Introduction** | | | |
| Background/rationale | 2 | Explain the scientific background and rationale for the investigation being reported | 3-5 #54-77 |
| Objectives | 3 | State specific objectives, including any prespecified hypotheses | 5 #113-122 |
| **Methods** | | | |
| Study design | 4 | Present key elements of study design early in the paper | 5,6 #123-155 |
| Setting | 5 | Describe the setting, locations, and relevant dates, including periods of recruitment, exposure, follow-up, and data collection | 5,6 #123-159 |
| Participants | 6 | RECORD 6.1: The methods of study population selection (such as codes or algorithms used to identify subjects) should be listed in detail. If this is not possible, an explanation should be provided. | 5,6 #124-155; 16,17 #367-393 |
| | | RECORD 6.2: Any validation studies of the codes or algorithms used to select the population should be referenced. If validation was conducted for this study and not published elsewhere, detailed methods and results of how study participants were identified from medical records should be provided. | 16 #367-374 |
| | | RECORD 6.3: If the study involved linkage of databases, consider use of a flow diagram or other graphical display to demonstrate the data linkage process, including the number of individuals with linked data at each stage. | not applicable |
| Variables | 7 | A complete list of codes and algorithms used to classify exposures, outcomes, confounders, and effect modifiers should be provided. If these cannot be reported, an explanation should be provided. | 16 #367-374 |
| Data sources/ measurement | 8* | For each variable of interest, give sources of data and details of methods of assessment (measurement). Describe comparability of assessment methods if there is more than one group | 6 #140-159 |
| Bias | 9 | Describe any efforts to address potential sources of bias | 16 #367-374 |
| Study size | 10 | Explain how the study size was arrived at | 8 #194 |
| Quantitative variables | 11 | Explain how quantitative variables were handled in the analyses. If applicable, describe which groupings were chosen and why | 6 #147-155 |
| Statistical methods | 12 | (*a*) Describe all statistical methods, including those used to control for confounding | 6,7 #160-178 |
| | | (*b*) Describe any methods used to examine subgroups and interactions | 6,7 #161-169 |
| | | (*c*) Explain how missing data were addressed | not applicable |
| | | (*d*) *Case-control study*—If applicable, explain how matching of cases and controls was addressed<br>*Cross-sectional study*—If applicable, describe analytical methods taking account of sampling strategy | not applicable |
| | | (*e*) Describe any sensitivity analyses | 6,7 #160-178 |

| | | | |
|---|---|---|---|
| Data access and cleaning methods | 12.1 | Authors should describe the extent to which the investigators had access to the database population used to create the study population. RECORD | 6 #138-146 |
| | 12.2 | Authors should provide information on the data cleaning methods used in the study. | 6 #146-155 |
| Linkage | 12.3 | State whether the study included person- level, institutional-level, or other data linkage across two or more databases. The methods of linkage and methods of linkage quality evaluation should be provided. | not applicabl e |
| Results | | | |
| Participants | 13* | Describe in detail the selection of the persons included in the study (i.e., study population selection), including filtering based on data quality, data availability, and linkage. The selection of included persons can be described in the text and/or by means of the study flow diagram. | 7 #181-18 6, 8 #194 |
| Descriptive data | 14* | (a) Give characteristics of study participants (eg demographic, clinical, social) and information on exposures and potential confounders | 7 #181-186 |
| | | (b) Indicate number of participants with missing data for each variable of interest | not applicable |
| | | (c) *Cohort study*—Summarise follow-up time (eg, average and total amount) | not applicable |
| Outcome data | 15* | *Cohort study*—Report numbers of outcome events or summary measures over time | not applicable |
| | | *Case-control study*—Report numbers in each exposure category, or summary measures of exposure | not applicable |
| | | *Cross-sectional study*—Report numbers of outcome events or summary measures | not applicable |
| Main results | 16 | (*a*) Give unadjusted estimates and, if applicable, confounder-adjusted estimates and their precision (eg, 95% confidence interval). Make clear which confounders were adjusted for and why they were included | 7-12 #179-254 |
| | | (*b*) Report category boundaries when continuous variables were categorized | 7-12 #179-254 |
| | | (*c*) If relevant, consider translating estimates of relative risk into absolute risk for a meaningful time period | |
| Other analyses | 17 | Report other analyses done—eg analyses of subgroups and interactions, and sensitivity analyses | 9-10 #206-222 |
| **Discussion** | | | |
| Key results | 18 | Summarise key results with reference to study objectives | 12-13 #255-289 |
| Limitations | 19 | Discuss the implications of using data that were not created or collected to answer the specific research question(s). Include discussion of misclassification bias, unmeasured confounding, missing data, and changing eligibility over time, as they pertain to the study being reported. | 16-17, #367-3 93 |
| Interpretation | 20 | Give a cautious overall interpretation of results considering objectives, limitations, multiplicity of analyses, results from similar studies, and other relevant evidence | 14-17, #324-393 |
| Generalizability | 21 | Discuss the generalisability (external validity) of the study results | 17 #395-399 |
| **Other information** | | | |
| Funding | 22 | Give the source of funding and the role of the funders for the present study and, if applicable, for the original study on which the present article is based | not applicable |
| Accessibility of protocol, raw data, and programming code | 23 | Authors should provide information on how to access any supplemental information such as the study protocol, raw data, or programming code. | 26 #606 |

Benchimol EI, Smeeth L, Guttmann A et al, RECORD Working Committee. **The REporting of studies Conducted using Observational Routinely-collected health Data (RECORD) statement.** PLoS Med. 2015 Oct 6;12(10):e1001885.
